# Medical education using minimal technology: achieving professional development

**DOI:** 10.1080/10872981.2019.1622365

**Published:** 2019-05-23

**Authors:** Karen A. Friedman, Saori W. Herman, Alice Fornari

**Affiliations:** a Department of Medicine, Donald and Barbara Zucker School of Medicine at Hofstra/Northwell, Manhasset, NY, USA; b Science Education, Occupational Health, Family Medicine Associate Dean, Educational Skills Development, Donald and Barbara Zucker School of Medicine at Hofstra/Northwell Vice President, Faculty Development, Northwell Health Director, MS degree, Health Professions Pedagogy and Leadership (HPPL), Hofstra University, Hempstead, NY, USA

**Keywords:** Medical education, journal club, interprofessional, on-line

## Abstract

**Background**: Traditional journal clubs have been limited by the geography of participants. Web based modalities and social media platforms are now being used to bridge this barrier. Medical education journal club, given the diversity of its community would lend well to these platforms. To date there is very little published regarding online medical education journal clubs.

**Objective**: To bridge geographical barriers; enhance interdisciplinary and interprofessional discussion and collaboration; and to provide opportunities for continuing medical education and faculty development; a monthly synchronous medical education journal club was created for faculty.

**Design/Methods**: From April 2015 to November 2016, 11 online journal clubs were held for the faculty at Northwell Health and the Barbara and Donald Zucker School of Medicine at Hofstra/Northwell (Zucker SOM). All articles picked were relevant to medical education and participants were from multiple disciplines.

**Results**: 74% of respondent participants agreed that the content covered during the sessions would positively impact personal and/or professional life and 58% of respondents reported that their overall knowledge/skill level changed positively.

**Conclusions**: On-line medical education journal club can provide a valuable opportunity for continuing education and faculty development for both the participant and the presenter.

## Introduction

Since their advent in 1875 by Sir William Osler at McGill University [1], journal clubs have played an integral role in medical education, especially that of graduate medical education (GME). The initial intent of journal clubs was to provide physicians with an opportunity to keep up-to-date with current research[1]. More recently, the clubs have provided a setting to facilitate discussion of topics such as critical appraisal, research design, and statistics[1].

One major drawback of journal clubs is the inability of participants to attend in-person meetings, particularly when they are not in the same location[2]. This issue can be remedied with online web-based modalities which allow participants to attend the meeting from any location either synchronously or synchronously [3–6]. This also allows for an expanded presenter pool. Furthermore, the recordings and chat dialog from the meetings can be digitally archived for future consumption and dissemination. Journal clubs utilizing social media platforms such as Twitter, blogs, and Google Hangout have shown success in terms of engagement across multiple disciplines [7–14]. Chan et al describe in the literature ten steps for creating an online journal club[15].

There is a dearth in the literature regarding interdisciplinary topics in journal clubs, most notably the topic of medical education. Despite the growth of medical education as a science, there are few published reports on journal clubs – in-person or online – dedicated to this topic. Medical education journal clubs can allow participants to keep up to date with current health sciences education literature as well as foster education research[16].

To bridge geographical barriers; enhance interdisciplinary and interprofessional discussion and collaboration; and to provide opportunities for continuing medical education and faculty development; a monthly synchronous medical education journal club was created for the faculty at Northwell Health and the Zucker School of Medicine (SOM)[17]. The purpose of this article is to discuss the creation, implementation, and assessment of this online medical education journal club (MedOnline).

## Methods

Starting from April 2015, synchronous online journal club meetings have been held on a monthly basis with the exception of the summer months (June, July, and August) and December. Meetings are scheduled on a weekday and are one-hour in length[17]. Advertising for the MedOnline is done through mass emailing of all the medical staff and faculty of affiliated hospitals, GME committees, and the School of Medicine. Participants who register through the Continuing Medical Education (CME) Tracker are given CME credit for attending the live MedOnline meetings only.

All meetings are hosted using the online web conferencing platform, GoToMeeting. The platform tools that are used most often are: the webcam, recording functions, screen sharing, and the chat box. All journal club meetings are recorded and available on the Zucker School of Medicine’s Faculty Development site[17].

Faculty presenters, spanning multiple disciplines are chosen based on interest in teaching medical education topics. Faculty development for the presenter includes guiding the format of the PowerPoint presentation, reviewing and editing the presentation, and moderating the discussions during the interactive portion of the presentation. Formative data is collected via a survey at the end of each meeting.

Two medical education articles based on a specific theme are selected for each session by the faculty presenter and the Assistant Vice President of Faculty Development (Table 1). The presenter deconstructs the articles and provides probing questions between each article to initiate conversation[17].

**Table 1.** Medical education online journal club topics.

Date	Presenter	Topic
September 2015	Saima Chaudhry, MD, MSHS -Internal Medicine	Reform of Didactics in GME
October 2015	Adam Aponte, MD, MSc, FAAP-Pediatrics	Diversity in Academic Environments
November 2015	Ronald Kanner, MD -Neurology	Evidence Based Medicine
January 2016	Steven Rubin, MD -Family Practice Wendy Herman MLIS, AHIP -Librarian	Unprofessional Behavior
February 2016	Michael Cassara, DO, MSEd, FACEP Wendy Herman MLIS, AHIP -Librarian	Simulation
March 2016	Karen Friedman, MD -Internal Medicine Wendy Herman MLIS, AHIP -Librarian	Patient Satisfaction
April 2016	Jennifer Verbsky, MD -Internal Medicine Wendy Herman MLIS, AHIP -Librarian	Coaching in Medical Education
May 2016	David Marcus, MD – Emergency Medicine Wendy Herman MLIS, AHIP -Librarian	Social Media’s application to Medical Education across the continuum
September 2016	R. Ellen Pearlman MD -Internal Medicine Wendy Herman MLIS, AHIP -Librarian	Unprofessional Behavior
October 2016	John Q. Young MD MPP PhD – Psychiatry Wendy Herman MLIS, AHIP -Librarian	Cognitive Load Theory
November 2016	Andrew C. Yacht MD -Internal Medicine Wendy Herman MLIS, AHIP -Librarian	Burnout

Beginning in January 2016, a medical librarian was added as a regular contributor to the club. The librarian covers the limitations, search methodology and bibliometrics of each article. Bibliometrics is a statistical measure of written publications which provides a general idea of the articles’ impact and influence. Traditional metrics captured by Web of Science and Google Scholar as well as alternative metrics such as social media site mentions, newspaper articles, and government reports captured by Altmetrics are discussed during the MedOnline[17].

The process of setting up a MedOnline requires logistical planning and support. One administrative support person covered the registration process, marketing, dissemination of email reminders, and any other housekeeping items. A technical support person from the Zucker SOM provided assistance on how to use the web conferencing software; acted as the point-person for troubleshooting technology-related issues before, during and after webinars; and records, downloads, and posts webinars. The Vice President for faculty development at the Zucker SOM provides assistance for the PowerPoint presentation, reviewing and editing of the presentation, moderating the discussions during the interactive question(s) portion of the presentation, and acting as a champion for the club on a monthly basis. This project was reviewed and deemed not to be research as it is a continuing professional development program by the IRB at Northwell Health.

## Results

**Figure 1.**
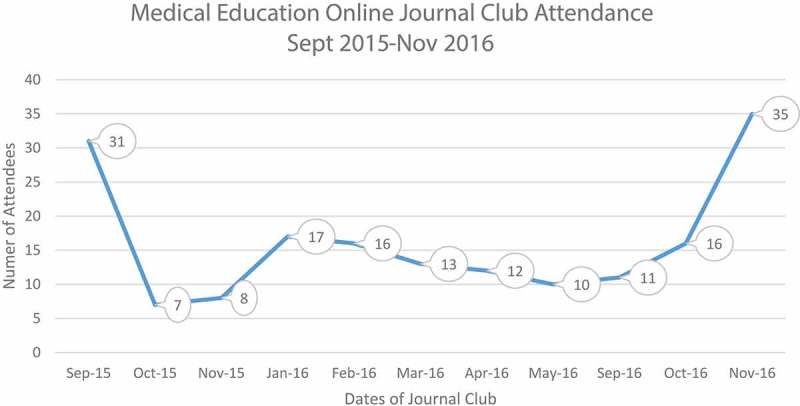
Medical education online journal club attendance.

**Figure 2.**
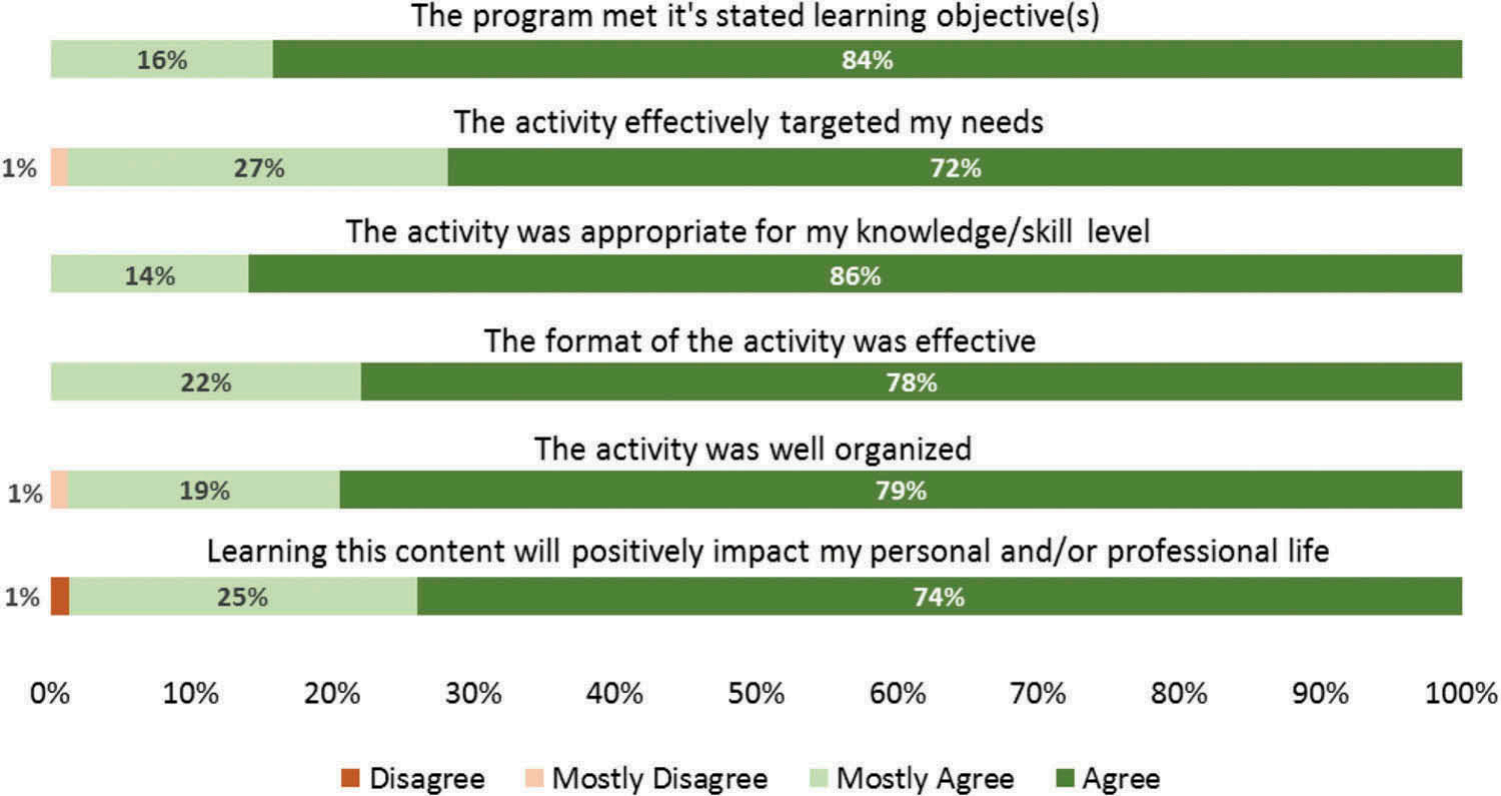
The distribution of responses to each of the evaluation questions as a pooled percentage.

On average, there were 16 participants per session over the 11 sessions (Figure 1). The participants were a mix of clinicians, librarians, and educators. The type of articles reviewed spanned a range of medical education topics (Table 1). The survey sent at the end of every session had a mean response rate of 49%. Figure 2 shows the distribution of responses to each of the evaluation questions as a pooled percentage. Almost all respondents agreed or mostly agreed with each statement. The respondents agreed that the program met its learning objectives (84%), the activity targeted their needs (72%) and was appropriate for their knowledge/skill level (86%). Regarding the logistics of the Journal Club, respondents agreed that the format of the activity was effective (78%) and well organized (79%). Overall, they agreed that the content covered during the sessions would positively impact personal and/or professional life (74%). Fifty eight percent of respondents reported that their overall knowledge/skill level had improved after the session and 60% reported that they intended to make changes in their clinical and/or educational environment as a result of the sessions. Informal comments about the librarian’s role from both participants and presenters have been positive.

## Discussion

Web based modalities have allowed journal clubs to enter the 21^st^ century[7]. By removing the geographical barrier, a larger array of participants can be reached. This is particularly useful when discussing medical education topics as the participants are often from different disciplines and locations. The addition of a medical librarian allowed for an expanded discussion of the literature and an introduction to traditional and alternative bibliometrics. One newly published article on patient experiences did not have any records within traditional bibliometric platforms such as Web of Science or Google Scholar. However, Altmetrics captured a great number of activity – mostly social media mentions (e.g. tweets) on the article. This example captured the attention of the participants as it provided a concrete example of how quickly information travels within non-traditional formats and how this can potentially affect the article’s impact and influence.

The first limitation noted was whether the participants thoroughly read the articles before the meeting. Lack of familiarity with the article can affect the depth and quality of the discussion. Second, it is unclear how many participants are actively listening during the meeting. Third, more time is needed for interactive discussion. Some themes require more time than the scheduled one hour. Finally, the survey needs to potentially be revised to ensure that the journal club’s objectives are being met and that the effectiveness of the meetings is being captured properly.

In the future, we hope to increase attendance by including other social media channels in the advertising process. We also hope to improve participation using the KeyLIME format of the Royal College of Physicians and Surgeons of Canada with the addition of a discussant alongside the presenter[18]. Similarly, we hope to explore ways to continue the interactive discussions beyond the webinar, using either social media channels or other web-based modalities. We would also like to add an interprofessional focus to the experience. Currently the majority of participants are physicians and librarians but we would like to encourage participation of other health professionals. Finally, we hope to have a better understanding of our medical educators’ topics of interest so that we can continue to provide interesting themes for each meeting.

## Conclusion

The MedOnline has been well received by participants and faculty presenters. The positive feedback as well as the steadily increasing number of participants and returning participants indicates the club’s success at Northwell Health and the Zucker School of Medicine. The MedOnline provides a valuable opportunity for continuing education and faculty development for both the participant and the presenter.
